# Two Mitochondrial Barcodes for one Biological Species: The Case of European Kuhl's Pipistrelles (Chiroptera)

**DOI:** 10.1371/journal.pone.0134881

**Published:** 2015-08-04

**Authors:** Tommy Andriollo, Yamama Naciri, Manuel Ruedi

**Affiliations:** 1 Muséum d’histoire naturelle de la Ville de Genève, BP 6434, 1211 Geneva 6, Switzerland; 2 Université de Genève, Faculté des Sciences, Section de biologie, 1211 Geneva 4, Switzerland; 3 Conservatoire et Jardin botaniques de la Ville de Genève and University of Geneva, BP 60, 1292 Chambésy, Geneva, Switzerland; University of Innsbruck, AUSTRIA

## Abstract

The Kuhl’s pipistrelle (*Pipistrellus kuhlii*) is a Western Palaearctic species of bat that exhibits several deeply divergent mitochondrial lineages across its range. These lineages could represent cryptic species or merely ancient polymorphism, but no nuclear markers have been studied so far to properly assess the taxonomic status of these lineages. We examined here two lineages occurring in Western Europe, and used both mitochondrial and nuclear markers to measure degrees of genetic isolation between bats carrying them. The sampling focused on an area of strict lineage sympatry in Switzerland but also included bats from further south, in North Africa. All individuals were barcoded for the COI gene to identify their mitochondrial lineages and five highly polymorphic microsatellite loci were used to cluster them according to their nuclear genotypes. Despite this low number of nuclear markers, all North African nuclear genotypes were grouped in a highly distinct subpopulation when compared with European samples sharing the same mitochondrial barcodes. The reverse situation prevailed in Switzerland where bats carrying distinct barcodes had similar nuclear genotypes. There was a weak east/west nuclear structure of populations, but this was independent of mitochondrial lineages as bats carrying either variant were completely admixed. Thus, the divergent mitochondrial barcodes present in Western Europe do not represent cryptic species, but are part of a single biological species. We argue that these distinct barcodes evolved in allopatry and came recently into secondary contact in an area of admixture north of the Alps. Historical records from this area and molecular dating support such a recent bipolar spatial expansion. These results also highlight the need for using appropriate markers before claiming the existence of cryptic species based on highly divergent barcodes.

## Introduction

DNA barcoding offers an aid for the identification and discovery of cryptic lineages by using a standardized mitochondrial gene, the cytochrome *c* oxidase subunit I (COI) for most animals [1]. The barcode approach is particularly effective in differentiating species in highly diversified groups that are difficult to identify using classical morphologic approaches (e.g. [2, 3, 4]). One such group is the Chiroptera, which includes many closely related species that may share very similar morphologies and ecologies [5]. Such morphological similarities may result in overlooked diversity, as it has been suggested in European taxa such as the vespertilionids, in which the species number has increased by almost 50% since the emergence of DNA sequencing in taxonomy [6].

The Kuhl’s pipistrelle, *Pipistrellus kuhlii* (Kuhl, 1817), is perhaps the commonest bat species in the Mediterranean region [7, 8]. It apparently recently extended its range by recolonizing more temperate regions of Europe [9, 10]. Despite being a common species, its systematics remains unclear regarding the taxonomy of some peripheral populations. For instance, an eastern form, *P*. *k*. *lepidus* Blyth, 1845, and the Macaronesian endemic, *P*. *maderensis* (Dobson, 1878), are sometimes regarded as true species or as subspecies of the Kuhl’s pipistrelle [11, 12]. Likewise, a desert form living in arid areas of North Africa, *P*. *k*. *deserti* Thomas, 1915, has also been considered as a full species based on its distinct morphology, but nuclear [13] and mitochondrial [8] markers showed that this morphotype evolved multiple times in different desert regions from typical *P*. *kuhlii* populations and is now considered as a desert form of *P*. *kuhlii* [8, 13].

**Fig 1:**
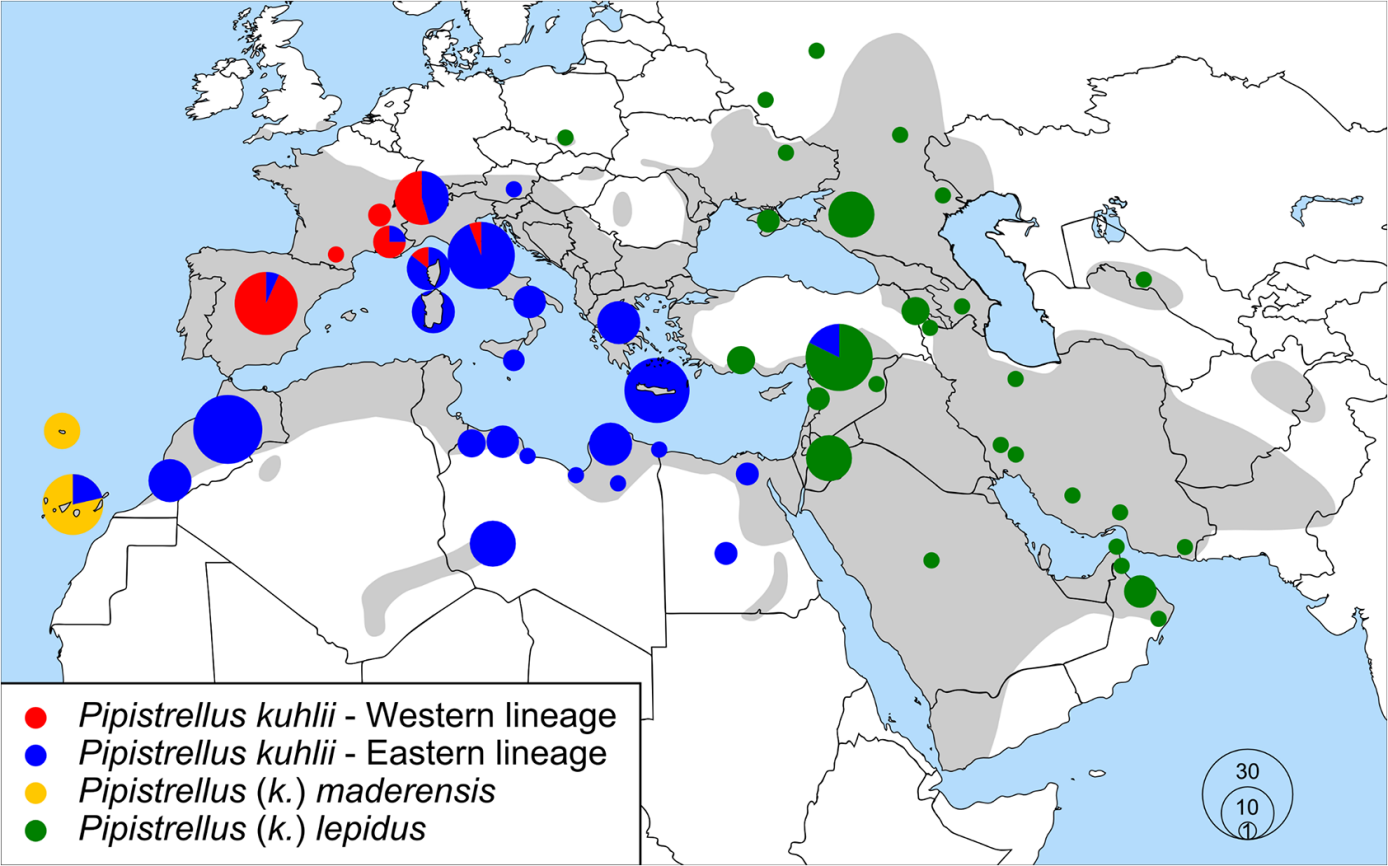
Distribution map of the four main mitochondrial lineages identified within the *P*. *kuhlii* species complex. In grey, the current distribution of this species complex based on Aulagnier et al. [23] and Benda et al. [24] and modified according to Dietz et al. [12] and Sachanowicz et al. [10]. Note that *deserti* is considered here as a desert morphoptype of *P*. *kuhlii* [13] and shares with the latter haplotypes of the Eastern lineage. Coloured dots represent the distribution of the main lineages identified from various mitochondrial markers (Mayer and von Helversen [25], Pestano et al. [14], Stadelmann et al. [26], Ibáñez et al. [17], Mayer et al. [6], García-Mudarra et al. [18], Evin et al. [15], Veith et al. [20], Galimberti et al. [19], Kruskop et al. [21], Bray et al. [27], Çoraman et al. [16], Jesus et al. [28], Benda et al. [13], Benda et al. [8], Bogdanowicz et al. [29]). The inset provides the colour legend for each lineage. Circle size reflects the approximate number of sampled specimens.

Several studies using distinct mitochondrial markers showed that lineages representing *P*. *maderensis* and *P*. *k*. *lepidus* were part of an unresolved polytomic tree containing other lineages of *P*. *kuhlii sensu stricto* and rendering the latter taxon paraphyletic (e.g. [14, 15, 16]). The genetic divergence between the main lineages in this complex is ca. 6% for cyt-*b* and ND1 genes [16, 17], and molecular surveys further showed that the two major lineages of *P*. *kuhlii s*.*s*. co-occur in large parts of Western Europe [17–21], including Corsica [15] (Fig 1). Individuals from both lineages have also been found in Northern Italy [15] near the type locality of *P*. *kuhlii s*.*s* (Trieste) [22]. One of these major continental lineages is largely restricted to regions west of the Alps (Fig 1) and will be referred hereafter as the “Western lineage”. The other major lineage appears to be rare in Western Europe, but more common east and south of the Alps and it is the only one existing throughout North Africa (Fig 1), including the *deserti* morphotype. This second lineage is called here the “Eastern lineage”. According to Çoraman et al. [16], this lineage is present as far east as along the southern coast of Turkey, but is largely replaced by the “*lepidus*” lineage east of the Balkans and over most of the Middle East.

Earlier studies based on mitochondrial markers claimed that “highly divergent mitochondrial lineages provide strong evidence for cryptic species diversity” [6], but no other data (morphological, ecological or nuclear markers) substantiate this hypothesis. Furthermore, due to the special mode of inheritance of the mitochondrial genome (i.e. transmitted clonally by females only, with no recombination), taxonomic conclusions based exclusively on this genome can be misleading [30–32].

Because of the important conservation issues associated with the existence of cryptic species [33], it is crucial to evaluate properly whether the divergent mitochondrial barcodes within *P*. *kuhlii* represent unsuspected biological species or not. In this study, we will focus on an area of sympatry in Switzerland, where bats of the Western and Eastern lineages meet and thus may interbreed, providing a unique opportunity to test their biological species status. For this purpose, we used the classical mitochondrial barcode (COI) to assign each bat to the corresponding lineage, and five independent nuclear markers to estimate their population structure and degree of reproductive isolation.

## Material and Methods

### Ethics statement

This work was exclusively based on existing tissues available in museum collections and thus required no ethical approval.

### Sampling and DNA extraction

**Fig 2:**
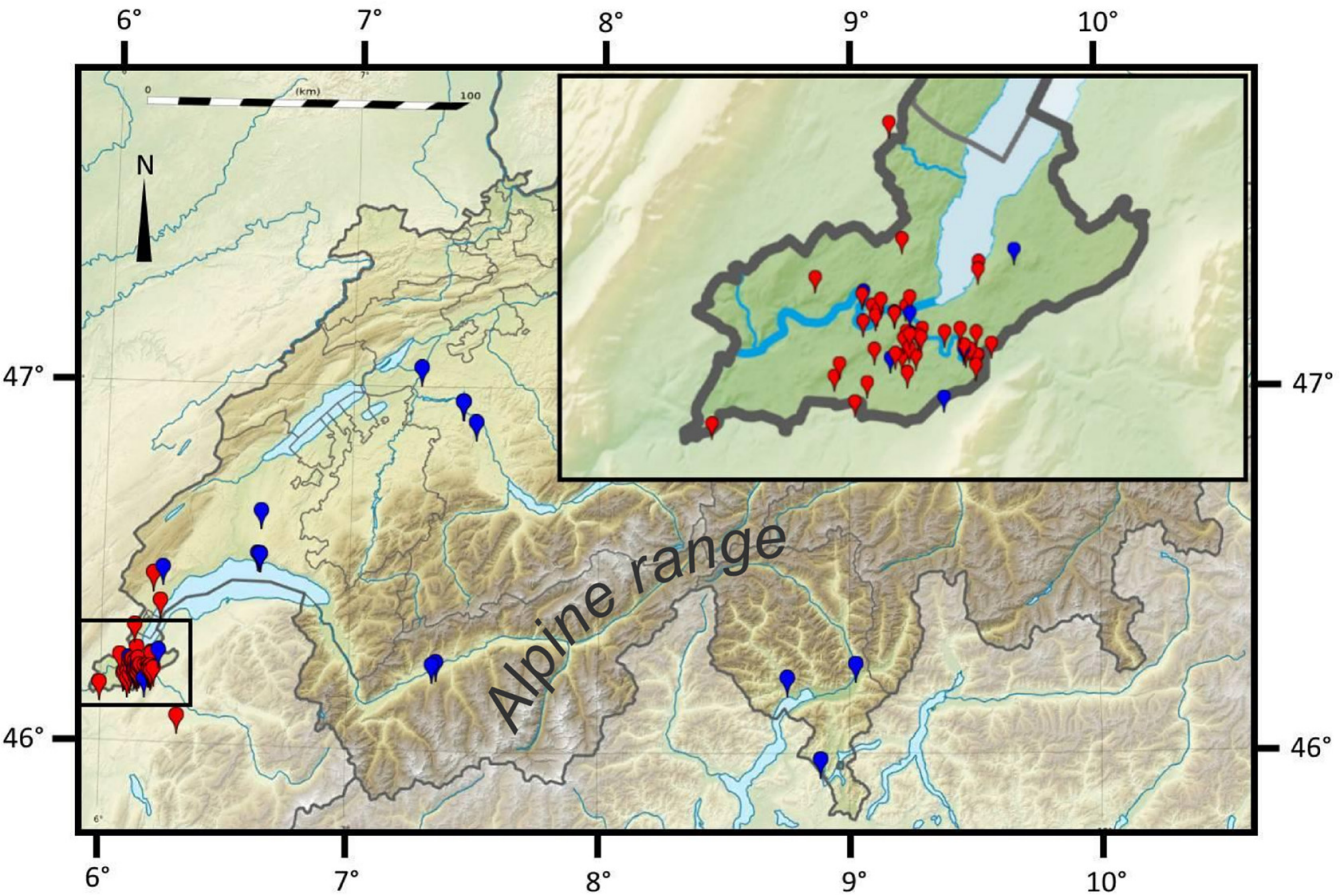
Sampling localities of *P*. *kuhlii* within Switzerland and neighbouring France. The inset provides a detailed map for the Geneva province. The sampling includes 79 specimens whose precise geolocation was available. Each individual is pictured as a dot, coloured in red or in blue depending on whether it belongs to the Western or to the Eastern mitochondrial lineage, respectively.

The current sampling included 101 bats morphologically identified as typical Kuhl’s pipistrelles [34] and 10 animals representing the *deserti* morphotype [13]. These samples were available from the frozen tissue collection associated to vouchered specimens held in the collections of the Natural History Museum of Geneva (MHNG, *n* = 65), the National Museum of Prague (NMP, *n* = 13), the Natural History Museum of Bern (NMBE, *n* = 4), the Natural History Museum of Lugano (MNHL, *n* = 3), the Stiftung für Fledermausschutz in Zürich (KOF, *n* = 10) and the Muséum national d’Histoire naturelle de Paris (MNHN, *n* = 16). These individuals came from Switzerland (*n* = 80), France (*n* = 18), Libya (*n* = 10) and Morocco (*n* = 3). A detailed figure of the Geneva region (Fig 2) illustrates the denser sampling used to measure the degree of reproductive isolation among *P*. *kuhlii* lineages.

Most of these specimens were recovered from "health care centres" where dead bats are frozen after an unknown exposure period at room temperatures. Thus several samples had highly degraded DNA. A fragment of breast muscle or a wing punch was taken from each frozen specimen and stored in pure ethanol at -20°C before analysis. DNA extractions were performed using the DNeasy Blood & Tissue Kit (Qiagen, Switzerland) according to the manufacturer’s instructions. The purified DNA was eluted in 200 μL of TE Buffer and stored at -20°C.

### mtDNA amplification and sequencing

Initially, we attempted to amplify the partial fragment of the cytochrome *c* oxidase I (COI) gene used as barcode [1] with the universal primers VF1d and VR1d [35]. However, because of the poor DNA quality of many samples, this large fragment (about 700 bp) did not amplify in several individuals. We therefore designed an alternative forward primer, COX1 Kuhl (5’-GCG CCT GAT ATA GCA TTN CCN CGA AT-3’), located about 230 bp downstream the start of the COI gene, in order to amplify a shorter fragment (about 470 bp). This mini-barcode amplified successfully in all remaining *P*. *kuhlii* samples and was sufficient to assign each individual to each of the two lineages. This newly designed primer proved to be also useful to amplify mini-barcodes in many other vespertilionids.

For both COI fragments, the amplifications were achieved in a 25 μL reaction volume, including 2.5 μL 10× Buffer (Qiagen, Switzerland), 1 μM MgCl_2_, 0.8 mM dNTP, 0.2 μM of each primer, 5 μL Q-Solution, 1 U Taq Qiagen and 3 μL of extracted DNA. The thermal cycling program started with an initial denaturation at 93°C for 3 min, followed by five touchdown cycles with 45 s denaturation at 93°C, 45 s annealing at a temperature set between 50 and 45°C (with a decreasing pitch of 1°C) and 1 min extension at 72°C, followed by 35 cycles with an annealing temperature set at 45°C and a final extension for 5 min at 72°C. The PCR product was purified using the High Pure PCR Product Purification Kit (Roche, Switzerland). For all full-length barcodes, PCR products were sequenced for both strands on a capillary sequencer ABI 3031 (Applied Biosystems), while mini-barcodes were only sequenced in the forward direction.

### mtDNA alignment and analyses

We aligned the COI sequences using the Clustal W alignment algorithm [36], and used criteria proposed by Song et al. [37] to exclude possible non-homologous sequences (Numts). None had double bands in the control agarose gels, ambiguous peaks in chromatograms, stop codons or indels in the translated sequences, and hence all sequences were assumed to be of mitochondrial origin.

As most of the previous studies used the cyt-*b* gene as mitochondrial marker, we sequenced the COI for six individuals whose cyt-*b* sequences were available on GenBank (see S1 File), in order to determine their corresponding lineage assignation to either Western or Eastern lineage. We downloaded 81 partial cyt-*b* sequences and aligned a fragment of 467 bp that was common to all these GenBank sequences (accession numbers are listed in S2 File). This alignment included 78 sequences representing the four main *P*. *kuhlii* lineages, in addition to two haplotypes representing *P*. *pygmaeus* and one of *P*. *pipistrellus* that were used as out-groups. Prior to analyses, redundant sequences were collapsed to unique haplotypes. A phylogeny was then reconstructed from this cyt-*b* alignment using the Bayesian inference method implemented in MrBayes [38, 39], under a GTR+G+I model of DNA evolution. Two independent MCMC chains of 10^6^ iterations were generated, and trees were sampled every 1000^th^ generation. The initial 250 sampled trees were discarded as burn-in. The six specimens sequenced for both COI and cyt-*b* were then located on the phylogeny to be assigned to one of the major European mitochondrial lineages.

The newly sequenced COI fragments of various lengths were aligned and, in order to include all individuals, we only considered the 384 bp that were in common between the full-length barcodes and the mini-barcodes. The different haplotypes were identified with DnaSP v5 [40]. Relationships among the mini-barcode haplotypes were represented on a median-joining network calculated using Network v. 4.6.1.2 [41, 42]. The demographic history of populations was explored with mismatch distributions calculated with Arlequin v. 3.5.1.2 [43] for the 84 individuals from Switzerland and neighbouring France (Fig 2). To estimate their demographic parameters, bats carrying one or the other mitochondrial lineage were analysed separately. MEGA5 [44] was used to calculated the K2P genetic distance between haplotypes and to estimate the transition/transversion bias (R) that best fits the data. These parameters were used to investigate and date the demographic history of each *P*. *kuhlii* lineage by testing the observed mismatch distributions against simulated (10^4^ iterations) populations experiencing a pure spatial expansion in Arlequin. The mode of the distribution, *τ*, was subsequently used to date the expansion event, using the formula *τ* = 2*Tμ* [45].

### Microsatellite amplification and allele calling

The following five primer pairs, known to amplify microsatellite loci in vespertilionids, were used to establish the multilocus nuclear genotype of each individual: EF6 (originally designed for *Eptesicus fuscus* [46]), Paur05 (*Plecotus auritus* [47]), Ppip05 and Ppip06 (*Pipistrellus pipistrellus* [48]) and L45 (*Pipistrellus abramus* [49]). Two further loci (Ppip 01 and Ppip04 [48]) were also assayed, but discarded because they showed clear evidence of null alleles (with frequencies > 5%), with several individuals failing to amplify these loci [50]. Amplifications were performed as simplex or multiplexed reactions as detailed in S3 File. PCR products were analysed together with a size standard on a Beckman Coulter GeXP Genetic Analysis System (Beckman Coulter, Switzerland). The allele calling was based on fragment size and further checked manually with the software GenomeLab (Beckman Coulter, Switzerland). Depending on the locus, 6 to 31 individuals were amplified, run multiple times and their alleles called independently to check the genotyping consistency [50]. Genotypes are available as supporting information (S4 File).

### Nuclear data analysis

We calculated the mean number of alleles per locus (*A*), observed heterozygosity (*H*_*O*_) and gene diversity ($\hat{H}$) in Arlequin and allelic richness (*Rs*) in Fstat 2.9.3 [51]. In order to identify the underlying population structure of the whole sample, we used a Bayesian clustering procedure implemented in Structure v. 2.3.4 [52, 53] with the admixture and correlated allele frequency models (*λ* = 1). This software uses a Markov Chain Monte Carlo (MCMC) algorithm and tries to cluster individuals in a given number of subpopulations (*K*) by minimizing departures from HWE and linkage disequilibrium among groups. We performed 20 independent runs for each of *K* = 1–8 and 110’000 MCMC generations. The initial 10% of generations were discarded as burn-in. The output files were then analysed with the online program Structure Harvester [54] in order to determine the most likely number of populations (*K*) underlying the datasets using Evanno’s criterion [55]. Results from the twenty runs were combined in CLUMPP [56], and the population structure was displayed as coloured bar plots with Distruct [57].

The precise geographical origin (i.e., capture site known at <1 km accuracy) was available for 108 out of the 111 genotyped specimens (see S1 File). We also performed a Geneland analysis [58] using R, in order to incorporate this geographic information into the inferred genetic structure. Moreover, among the several existing methods using geographical location in combination with genotypic data, Geneland is better suited in cases where population are not at demographic equilibrium [59], as it is expected in the expanding *P*. *kuhlii*. As for Structure, Geneland uses MCMC chains to cluster individuals in a number of subpopulations that are as close as possible of Hardy-Weinberg and linkage equilibria (HWLE). Five independent runs were performed for *K* = 1–8, using 10^6^ iterations, including a 10% burn-in period, a 100-fold thinning and a correlated allele frequency model. All runs gave comparable results, with the same clustering of individuals in a given *K* number of subpopulations. An Amova performed on the inferred clusters was run with Geneland to obtain fixation indices within (F_IS_) and between (F_ST_) the inferred subpopulations.

## Results

### Lineage assignment

**Fig 3:**
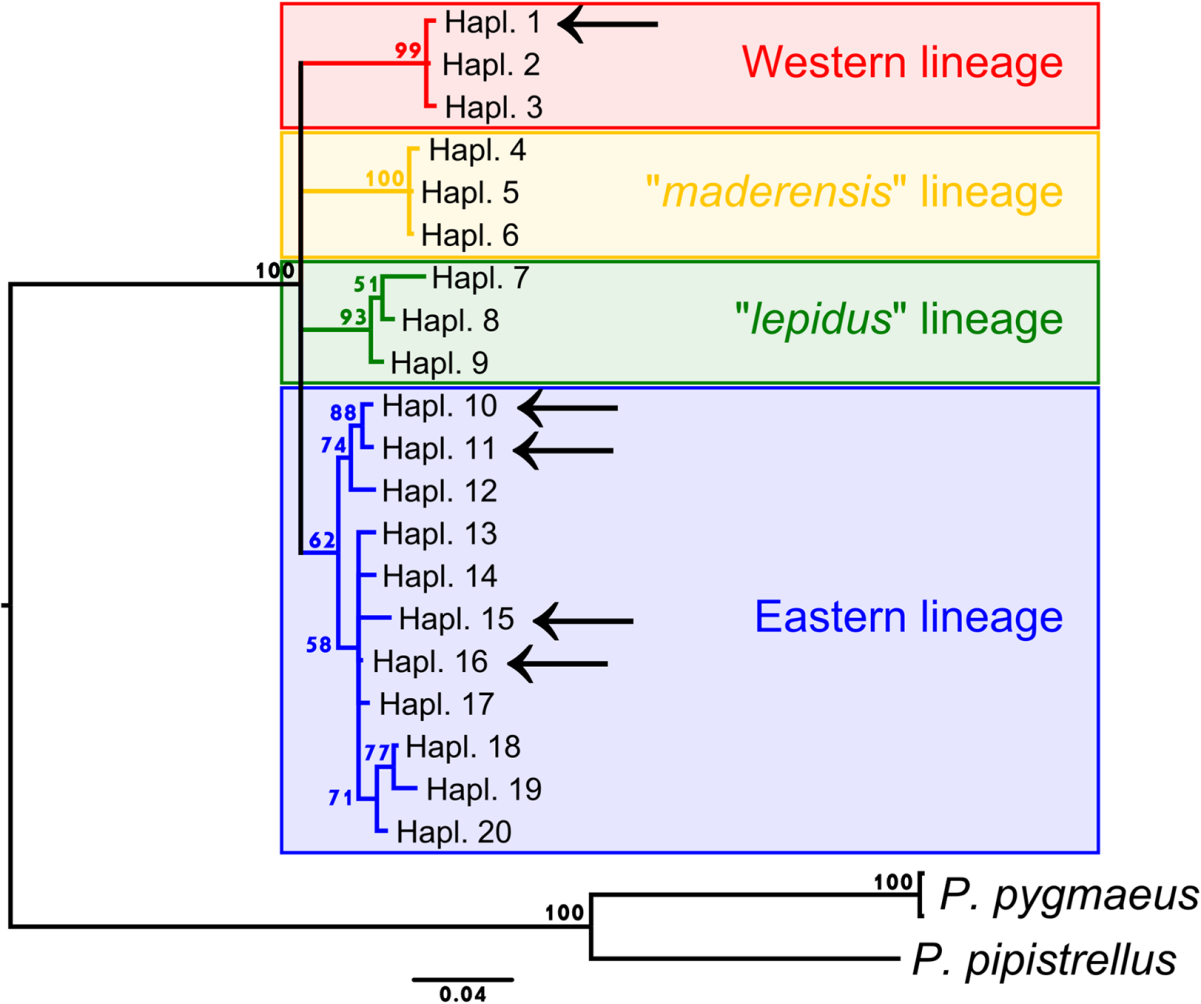
Phylogeny inferred by MrBayes from partial cyt-*b* sequences (467 bp). Coloured frames correspond to the four main mitochondrial lineages usually recognized within the *P*. *kuhlii* species complex. Posterior probabilities (PP) greater than 50% are indicated near the nodes. The position of the five cyt-*b* haplotypes observed in individuals also sequenced in this study for the COI gene are indicated with arrows. GenBank accession numbers for sequences used in this phylogeny can be found in S2 File.

**Fig 4:**
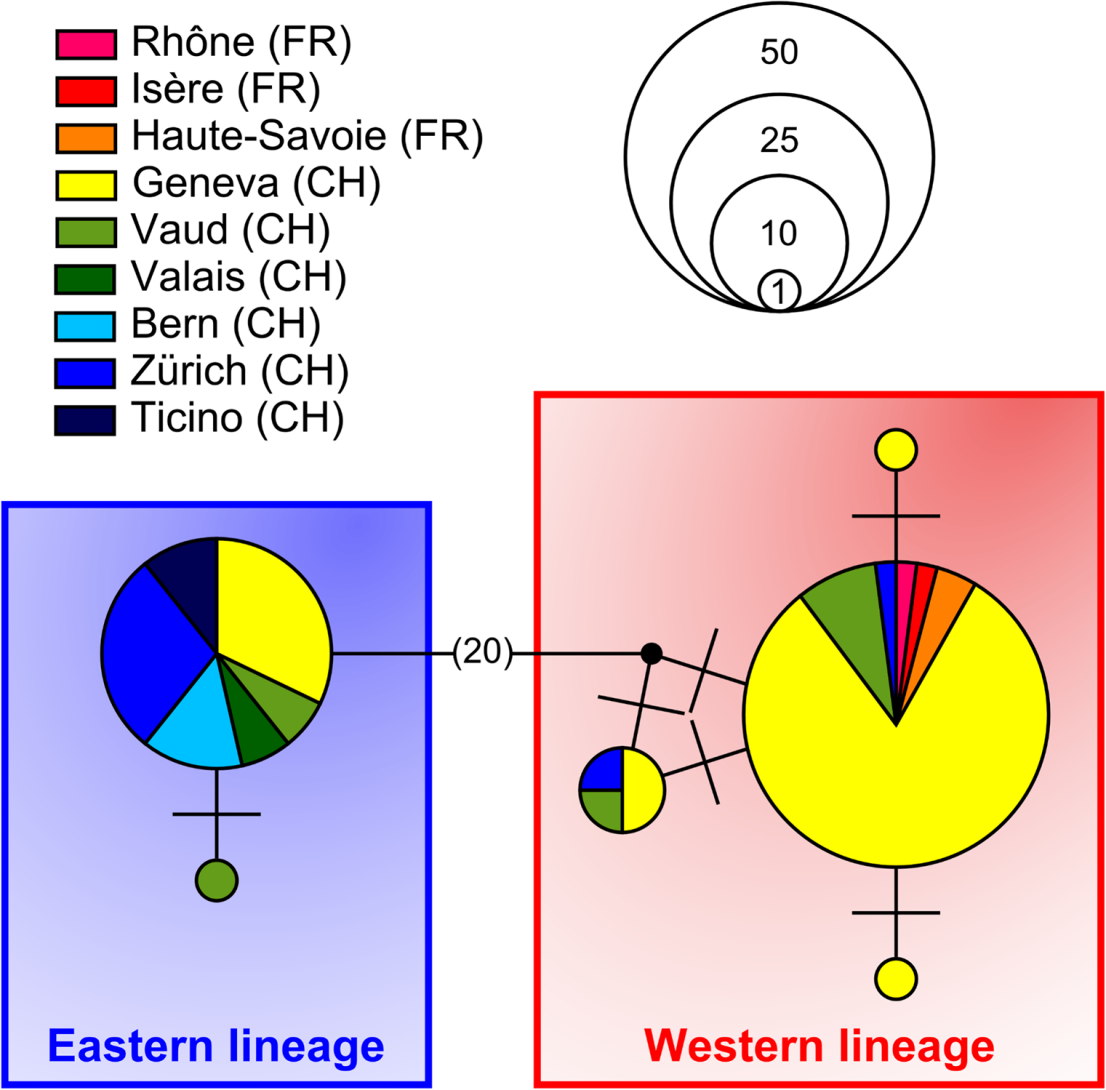
Median-joining network of COI haplotypes (384 bp) from Switzerland and neighbouring France. This network is restricted to 84 *P*. *kuhlii* that were sampled within a radius of 40 km, i.e. within flying distance for these bats. Each variant is represented as a circle, the area of which is proportional to the number of individuals sharing this haplotype. Transversal bars indicate one mutation between haplotypes and black dots symbolize the reconstructed missing haplotypes. Red and blue frames correspond to the Western and Eastern lineages, respectively.

The phylogeny inferred from partial cyt-*b* sequences showed no resolution (<50% posterior probability) for basal nodes within *P*. *kuhlii* (Fig 3). Although this analysis was not designed to provide a solid phylogenetic framework, the resulting tree indeed supported the four main lineages recovered in more comprehensive molecular surveys [16, 20]. The Eastern lineage was the least supported (62% posterior probability, PP), the other ones had PP over 90%. European individuals used in this study were either part of the Western or the Eastern lineages. None of them were related to the “*maderensis*” or to the “*lepidus*” lineages. Likewise, specimens from North Africa (including the *deserti* morphotype [8, 13]) belonged to the Eastern lineage. As any other Kuhl's pipistrelles sequenced only for the CO1 gene were either identical or within two mutations of the reference specimens (Fig 4), their lineage assignment was trivial.

### mtDNA dataset

Full-length COI barcodes (657 bp) were available for 37 individuals, while only mini-barcodes (384 bp) could be obtained for 71 additional bats. Three individuals from Morocco with known cyt-*b* were not COI-barcoded. The new sequences are available in the BOLD platform under accession numbers CHIAA001-15 to CHIAA098-15.

Focusing on the 84 bats sampled in Switzerland and neighbouring France (Figs 2 and 4), six distinct haplotypes were observed. Four of them belonged to the Western lineage (*n* = 55 individuals) and the remaining two were part of the Eastern lineage (*n* = 29 individuals) (Fig 4). These two major lineages differed from each other by a mean of 20 mutations, corresponding to a mean K2P genetic distance of 6.1%. This divergence is comparable to that reported in previous studies based on larger fragments of other mitochondrial genes, ND1 and cyt-*b* [6, 15–17]. Both haplogroups consisted of a main haplotype found in most bats, plus few haplotypes differing by one or two mutations (Fig 4). The Western lineage included all French (*n* = 14), most of the Genevan (*n* = 41) and few other Swiss individuals (*n* = 10), while the Eastern lineage was only composed of Swiss individuals (*n* = 29) from all over the country, including three from the Ticino province, south of the Alps (see S1 File for further details).

The transition/transversion bias estimated with Mega for this dataset was *R* = 5.46. Using this value to simulate mismatch distributions under a spatial expansion scenario, we could not reject this hypothesis for each of the two mitochondrial groups, with *p*-values of 0.32 and 0.24 for the Western and Eastern lineages, respectively. The modes of the mismatch distributions were also comparable for the Western (*τ* = 0.09267 [95% CI = 0.00001–2.04618]) and for the Eastern lineages (*τ* = 0.14595 [95% CI = 0.00001–0.79419]). To date these expansions, the mutation rate was calculated by combining the K2P distance for the 384 bp COI fragment with the divergence time estimated by Bray et al. [27] between the two main mitochondrial lineages, i.e. 1.8 mya, and a generation time of one year was assumed for the Kuhl's pipistrelle. Given the large confidence intervals (CI) associated to the estimation of *τ* and the incertitude of such calibrations, the estimated expansion time should be considered with caution, but both indicate a recent expansion, including present (*T*_*Western*_ = 7′118 years [95% = 0–157′157] and *T*_*Eastern*_ = 11′210 *years* [95% = 0–60′997]).

### Nuclear diversity

None of the five microsatellite loci essayed in the densely sampled population from the Geneva area deviated significantly (*P* > 0.05) from HWE expectations, indicating that no null or ghost allele were present in this subsample. The genetic diversity at these five loci was greater among European samples compared to North African ones, for both allelic richness and heterozygosity level, but sample sizes differed considerably (Table 1). In North African pipistrelles, one of the five loci (Ppip05) was fixed for a private allele (165 bp), and at a second locus (Paur05), 85% of individuals were homozygous for another private allele (227 bp). No significant departure from HWE was noticed either for European or North African populations, although this test is questionable when applied at this large geographical scale.

**Table 1:** Standard diversity indices calculated for five microsatellite loci genotyped in two subpopulations of *P*. *kuhlii*.

Population	*n*	Locus	*A*	*Rs*	*H*_*O*_	$\hat{H}$
Europe	95	EF6	12	11.9	0.76	0.79
		Paur05	9	8.9	0.74	0.79
		L45	13	12.8	0.78	0.88
		Ppip05	17	16.9	0.77	0.91
		Ppip06	14	14.0	0.84	0.89
		**Mean**	**13 ± 2.9**	**12.9 ± 2.9**	**0.78 ± 0.04**	**0.85 ± 0.06**
North Africa	13	EF6	8	8.0	0.62	0.80
		Paur05	3	3.0	0.15	0.15
		L45	8	8.0	0.77	0.87
		Ppip05	1	1.0	–	–
		Ppip06	9	9.0	0.69	0.83
		**Mean**	**7 ± 2.7**	**7 ± 2.7**	**0.56 ± 0.28**	**0.66 ± 0.34**

Number of alleles per locus (*A*), rarefied allelic richness (*Rs*) observed heterozygosity (*H*_*O*_) and gene diversity ($\hat{H}$) are given. Means are given with their standard deviation (SD).

### Structure

**Fig 5:**
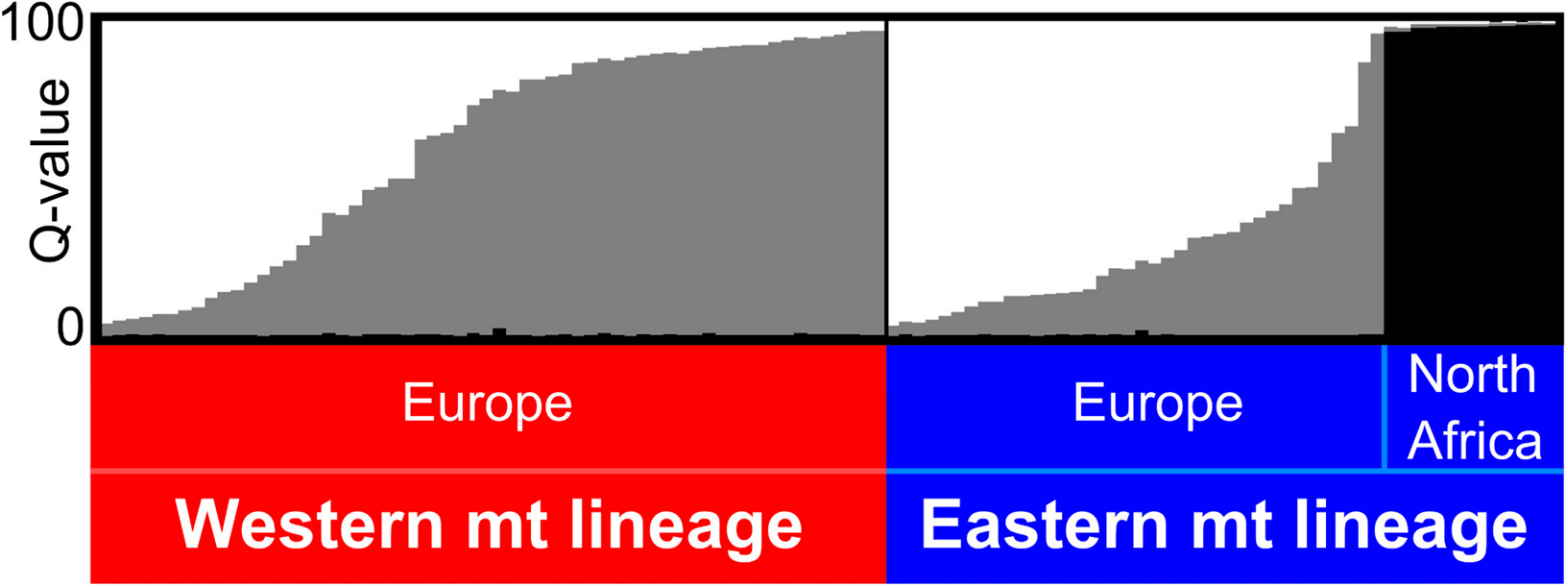
Population structure inferred for *K* = 3 by the Bayesian clustering analysis. The analysis was performed from alleles at five microsatellites for 111 *P*. *kuhlii*. An admixture model was used and 20 independent runs of Structure were performed in order to calculate a mean membership (Q-value) for each individual. Individuals are represented by vertical bars and sorted by mitochondrial lineage.

All 20 replicates of Structure analyses performed for eight different *K* values converged toward similar results. In all cases Evanno’s criterion designated *K* = 2 subpopulations as the most likely structure, and this corresponded to the sharp separation of the North African individuals from European ones. The *K* = 3 subpopulation subdivision (Fig 5) represents the next-preferred structure, and the one maximizing the likelihood. Graphs for *K* ranging from 2 to 8 are available as supporting information (S5 File). In addition to the distinct North African subpopulation, the *K* = 3 configuration distinguished two further subpopulations within Europe, but most individuals were admixed (Fig 5). By looking at the Q-values pertaining to each of these three groups, the North African samples (in black in the upper panel of Fig 5) were all clearly classified into one separated subpopulation ($\bar{Q_{black}}=0.98\pm 0.01$, *n* = 13). The genotypes from Europe formed a cline between pure white (in one subpopulation) to pure grey (in the other subpopulation) individuals, most of them being more or less admixed between these two extremes. If these European pipistrelles are sorted according to their main mitochondrial lineages (lower panel of Fig 5), the mean Q-value of individuals bearing the Western lineage is, on average ($\bar{Q_{grey}}=0.60\pm 0.34$, *n* = 60), slightly different from that of individuals pertaining to the Eastern one ($\bar{Q_{grey}}=0.28\pm 0.23$, *n* = 38). We tested the significance of this difference between mean Q-values in the Western *versus* Eastern lineages by resampling without replacement 10^5^ subgroups of 60 and 38 individuals, respectively, taken randomly in the pool of European genotypes, respectively. We then calculated the difference between Q-values of the two groups obtained with this non-parametric test, but none reached the actual $\Delta \bar{Q_{grey}}=0.327$ observed between the two groups sorted by mitochondrial lineages (*P*-value < 0.001). This indicates that bats belonging to either mitochondrial lineage tend to have a slightly different array of nuclear alleles.

### Geneland

**Fig 6:**
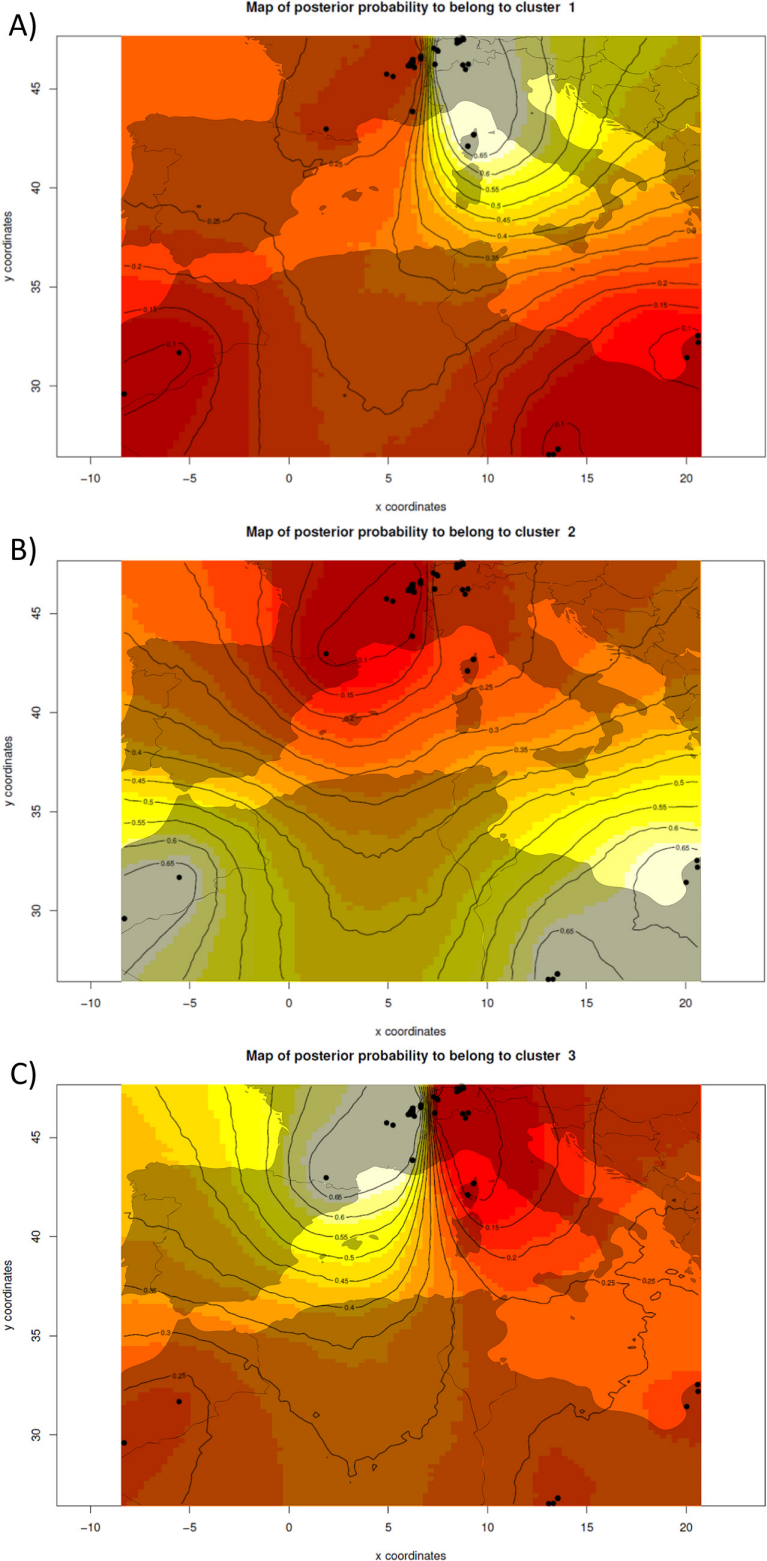
Maps of population membership probability to each of the three clusters inferred by Geneland. This analysis includes 108 Kuhl’s pipistrelles for which both multilocus genotypes and geolocation was available. The outlines of the European and North African landmasses are overlaid in grey to illustrate the approximate location of samples. Black dots identify the individuals’ location. x and y coordinates represent longitude and latitude, respectively.

Given the weak but noticeable overall differences in allelic composition at the five nuclear loci between individuals within Europe, we repeated this clustering analysis by integrating the geographical location into the assignment process, using Geneland. By taking the geographic information into account, the most likely number of populations appeared to be *K* = 3. As in Structure, the North African pipistrelles were clearly separated as one cluster, while the European samples were split into two parapatric subpopulations. The border between these two subpopulations runs across Switzerland (Fig 6), and separates individuals from mainland France and western Switzerland from those sampled in eastern Switzerland and Corsica. The map showing the membership probability of individuals to the three clusters inferred by Geneland illustrates this spatial pattern (Fig 6). The fixation indices within (F_IS_) and between (F_ST_) the three subpopulations are summarized in Table 2 and confirm the very strong structure due to the Mediterranean sea (F_ST_ = 24–31% of the total variance explained) compared to that found within Europe (F_ST_ lower than 5%). F_ST_ values further suggest that North African samples are more distantly related to those from Western Europe (F_ST_ = 31%) than to those from further east (F_ST_ = 24%).

**Table 2:** Fixation indices calculated for three inferred subpopulations of *P*. *kuhlii*.

**Subpopulation**	**East**	**West**	**North Africa**
East	4.6%		
West	4.4%	13.7%	
North Africa	23.7%	30.7%	16.5%

F_ST_ (lower triangle) and F_IS_ (diagonal) between and within the three subpopulations of *P*. *kuhlii* inferred by Geneland for five microsatellite loci. The “East” and the “West” subpopulations correspond to the two European subpopulations (Fig 6) but do not refer to mtDNA lineages.

## Discussion

Bats are a challenging group for mammal systematics, as phenotypical traits often provide limited criteria to discriminate closely related species in this diverse order [5]. Therefore, they represent a potential mine of cryptic species, and the barcode approach is a useful tool to identify possibly overlooked diversity. In several cases, the mitochondrial barcode analysis did help discovering new species, e.g. *Pipistrellus pygmaeus* [60] or *Myotis escalerai* [17], that were later confirmed as being biologically distinct using other approaches, including bioacoustics, morphology or nuclear genes [48, 61]. However, the typical barcode approach uses fragments of the mtDNA molecule that is inherited clonally from females and this technique is thus inappropriate to measure gene flow among individuals, i.e. the real criterion to discriminate biological species [62].

Four main mitochondrial lineages exist in the otherwise monophyletic *P*. *kuhlii* species complex [16]. The “*maderensis*” and “*lepidus*” lineages are distributed into islands of the Atlantic [14], or in Eastern Europe and the Middle East [16], respectively (Fig 1), and were not studied here. The other two major lineages live in Western Europe and North Africa, and although they have been found occasionally in close vicinity (Iberia, Corsica [15, 17]), no study attempted to determine whether bats carrying any of these distinct lineages could interbreed or not. Within a radius of less than 30 km in the Geneva region, i.e. within the breeding range of *P*. *kuhlii*, we showed, with a dense sampling, that bats carrying the Western and the Eastern lineages coexist in strict sympatry (Fig 2). Elsewhere, in eastern and southern Switzerland, sampling was more sparse, but showed that Kuhl’s pipistrelles were nearly exclusively represented by the Eastern lineage, as is the case at wider scales in Italy [19] or North Africa [8, 13] (Fig 1). Conversely, haplotypes from the Western lineage largely predominate over the rest of Western Europe (Fig 1), including the area of strict sympatry in Western Switzerland with a proportion of 4 to 1 (Fig 2).

Five nuclear microsatellites assayed in 111 Kuhl’s pipistrelles surveyed in (mainly central) Europe and North Africa perfectly discriminate individuals from these two regions (Fig 5), regardless their mitochondrial origin. The European samples include notably 16 Corsican specimens that are genetically indistinguishable from continental ones. This confirms the hypothesis suggested by Evin et al. [15] that Corsican *P*. *kuhlii* might have recently settled on the island, possibly from anthropogenic introductions from Italian or French source populations. This global, dual distinction of European versus North African populations of Kuhl’s pipistrelles also suggests that gene flow is reduced across the Mediterranean Sea for this bat, although much larger and denser sampling around the Mediterranean would be necessary to establish more precise patterns of gene flow in this area. Although five nuclear loci certainly give a poor representation of the whole genome, their strong ability to discriminate conspecific bats sharing identical mitochondrial barcodes across the Mediterranean (Figs 5 and 6, S5 File) further indicates that they provide enough resolution to identify putative distinct species, even closely related ones.

When focusing on the area of strict sympatry of both mitochondrial lineages in the Genevan area (Fig 2), no major genetic substructure was evidenced with classical Bayesian clustering analyses applied to nuclear genotypes (Fig 5). Nevertheless when bats from this region were artificially sorted according to their mtDNA barcode, the mean Q-value of group membership measured at the nuclear genotypes differ significantly (*P*-value < 0.001), suggesting some cryptic structure. Indeed, the use of geolocation for the clustering of individual nuclear genotypes suggests a notable (although weak) differentiation of two subpopulations within Switzerland (Fig 6). According to Geneland analysis, all sampled individuals in the Geneva area and mainland France are nuclearly distinct from those sampled further east (Fig 6A and 6C), while again those from North Africa constitute a third distinct subpopulation (Fig 6B). Thus, both nuclear and mitochondrial DNA co-vary with geography, but zones of admixture for these two classes of markers are not geographically concordant.

### Two mitochondrial barcodes for a single species

By considering results of nuclear markers, including the area of strict co-occurrence of mitochondrial lineages (Fig 2), Kuhl’s pipistrelles show complete admixture of genotypes in Switzerland and neighbouring France. Thus, although the mitochondrial lineages differ by up to 6.1% K2P distance at the COI gene, they clearly represent barcodes belonging to a single, interbreeding species. These two deeply divergent barcodes do not identify different biological species, contrary to general expectation [63]. This result also contradicts earlier claims that western European *P*. *kuhlii* might be composed of several cryptic species [6]. A similar approach, using a combination of nuclear and mtDNA markers, should be tested in the areas occupied by the other major lineages of *P*. *kuhlii* (“*maderensis*” and “*lepidus*”, Fig 1), which should be considered meanwhile as “unconfirmed cryptic species” (sensus Vieites et al. [64]).

In fact, the coexistence of two deeply divergent lineages within a single biological species is not uncommon in bats, although the reasons explaining this dual occurrence may vary. In *Eptesicus serotinus* for instance, ancient hybridization and gene introgression with *E*. *nilssonii* apparently explains why two, non-sister mitochondrial lineages exist today in different parts of its geographic range [65]. It is not likely the case in *P*. *kuhlii*, as all mtDNA variants evidenced so far in this species are monophyletic [16, 20] and are much more distantly related to other pipistrelle species. In stable, panmictic populations, persistent high effective population size (*N*_*e*_) could also enhance the prolonged survival of several ancient lineages within a species, as drift leading to their coalescence is a function of this parameter (*N*_*e*_ generations for an haploid genome) [66]. It is less likely to occur in Kuhl’s pipistrelles or more generally in populations that experienced fragmentation or demographic bottlenecks, e.g. during glacial periods [67, 68]. Finally, secondary admixture of isolated subpopulations can also explain the coexistence of divergent lineages within interbreeding populations [67, 68], such as in American *E*. *fuscus* [69]. The Kuhl’s pipistrelle may have retained divergent lineages in distinct compartment of the Mediterranean, that secondarily admixed in more northerly regions. Indeed, at least in areas north of the Alps, *P*. *kuhlii* populations have experienced a spectacular range expansion during the last decades [9, 10]. In the Geneva region studied here (Fig 2) for instance, this bat was virtually unknown until the late 1980s [70–73], while today it is one of the most common species breeding there (pers. obs.). Such a recent range expansion is confirmed by mismatch analyses, although large confidence intervals due to the shortness of the fragment analysed and the uncertainty associated with the molecular dating prevents meaningful resolution at this recent time scale. In addition, as *P*. *kuhlii* populations north of the Alps have likely experienced both demographic and spatial expansions, it is unclear whether the dates resulting from the mismatch analysis reflect either or both phenomenon in combination.

### Bipolar recolonization

An unsuspected aspect of this range expansion north of the Alps is that it resulted from a bipolar colonization. Indeed, both mtDNA (Figs 1 and 2) and nuclear data (Fig 6) show a similar longitudinal segregation of genotypes, suggesting that these two components are issued from distinct subpopulations of *P*. *kuhlii* even though more extensive genetic surveys would be necessary to determine the exact origins of those. Because of their very recent secondary contact, the signature of this dual origin is still significant, but barely measurable by classical methods of population structure analyses (Fig 5). Such a pattern of bipolar recolonization of populations north of the Alps corresponds to the ‘hedgehog paradigm’ [67], and has been evidenced in several other bat species, such as *Plecotus auritus* [74], *Barbastella barbastellus* [75] or *Pipistrellus pipistrellus* [76]. By contrast, *Myotis myotis* and other species of bats [74, 77–79] recolonized the same area of Switzerland from a single glacial refugium from the West with very limited transalpine gene flow [80] and do not exhibit strong phylogeographic structure in this region.

## Conclusion

Determining the biological species status of plants or animals presenting polymorphic characters has always been a challenge, especially if they are not amenable to easy breeding experiments [81]. Divergent barcodes represent such a form of polymorphism and a number of reviews have shown that integrative approaches combining independently inherited markers are necessary before reaching firm taxonomic conclusions [19, 64, 82]. Such integrative approaches are important as the conservation issues associated with the discovery of new species are becoming challenging for the protection of biodiversity. The use of multiple independent molecular markers also provides different, more complete, insights into the evolution of populations [79]. Beyond the resolution of the biological status of the Eastern and Western mitochondrial lineages in *P*. *kuhlii*, the distinctness of the North African subpopulation, compared with the European ones, is unexpected and can be of importance for conservations purposes. However all other major components of its entire distribution range need to be addressed, and probably with more nuclear markers, before being able to fully understand the evolutionary or taxonomic importance of these findings.

## Supporting Information

S1 File Sampling informations. (DOCX)

S2 File GenBank accession numbers for the cyt-*b* sequences used in the Fig 3. (DOCX)

S3 File Microsatellite primer informations and multiplexes. (DOCX)

S4 File Multilocus genotypes of all specimens. (DOCX)

S5 File Population structure inferred for K = 2 to 8 by the Bayesian clustering analysis. (DOCX)

S6 File Raw trace files for 545 fragment analysis runs. (ZIP)
