# Supplementary material for: Two Mitochondrial Barcodes for one Biological Species: The Case of European Kuhl's Pipistrelles (Chiroptera)
Source: PLoS One. 2015 Aug 4;10(8):e0134881. doi: 10.1371/journal.pone.0134881 (PMC4524706; doi:10.1371/journal.pone.0134881)
Supplement: S1 File — (DOCX) [file pone.0134881.s001.docx]

S1 File. Sampling informations. List of the 111 vouchered samples of Kuhl’s Pipistrelles with their approximate geographic location, voucher number (if any), and field number. The last two columns list the corresponding numbers in BOLD and GenBank where sequences of COI and cyt-*b*, respectively, have been deposited. The following acronyms were used: MHNG – Natural History Museum of Geneva; NMP – National Museum of Prague; NMBE – Natural History Museum of Bern; MNHL – uncatalogued specimens from the cantonal Museum of Natural History of Lugano; MNHN – Allowen Evin’s uncatalogued specimens deposited at the Muséum national d’Histoire naturelle de Paris.

| Subspecies | Country | Province | City and location | Latitude | Longitude | Scientific number | Field number | COI  BOLD ID | cyt-*b* GenBank number | |
| --- | --- | --- | --- | --- | --- | --- | --- | --- | --- | --- |
| *P. k. kuhlii* | FR | Alpes-de-Haute-Provence | Verdon | 43.847837 | 6.219277 | MNHN --- | 321_122386 | CHIAA091-15 | |  |
| *P. k. kuhlii* | FR | Alpes-de-Haute-Provence | Verdon | 43.847837 | 6.219277 | MNHN --- | 323_122384 | CHIAA093-15 | |  |
| *P. k. kuhlii* | FR | Alpes-de-Haute-Provence | Verdon | 43.847837 | 6.219277 | MNHN --- | 324_122385 | CHIAA094-15 | |  |
| *P. k. kuhlii* | FR | Ariège | Laroque-d'Olmes | 42.966667 | 1.866667 | MNHN --- | 322_124776 | CHIAA092-15 | |  |
| *P. k. kuhlii* | FR | Haute-Corse | Saint-Florent, préau de l'école | 42.683333 | 9.300000 | MNHN --- | 306_STF20 | CHIAA083-15 | |  |
| *P. k. kuhlii* | FR | Haute-Corse | Saint-Florent, préau de l'école | 42.683333 | 9.300000 | MNHN --- | 307_STF21 | CHIAA084-15 | |  |
| *P. k. kuhlii* | FR | Haute-Corse | Saint-Florent, préau de l'école | 42.683333 | 9.300000 | MNHN --- | 314_STF24 | CHIAA085-15 | |  |
| *P. k. kuhlii* | FR | Haute-Corse | Saint-Florent, préau de l'école | 42.683333 | 9.300000 | MNHN --- | 313_STF25 | CHIAA086-15 | |  |
| *P. k. kuhlii* | FR | Haute-Corse | Saint-Florent, préau de l'école | 42.683333 | 9.300000 | MNHN --- | STF26 | CHIAA087-15 | |  |
| *P. k. kuhlii* | FR | Haute-Corse | Saint-Florent, préau de l'école | 42.683333 | 9.300000 | MNHN --- | STF27 | CHIAA088-15 | |  |
| *P. k. kuhlii* | FR | Haute-Corse | Saint-Florent, préau de l'école | 42.683333 | 9.300000 | MNHN --- | STF28 | CHIAA089-15 | |  |
| *P. k. kuhlii* | FR | Haute-Corse | - | 42.100000 | 9.000000 | MNHN --- | 346_PAI1 | CHIAA096-15 | |  |
| *P. k. kuhlii* | FR | Haute-Corse | - | 42.100000 | 9.000000 | MNHN --- | FLO1 | CHIAA097-15 | |  |
| *P. k. kuhlii* | FR | Haute-Corse | - | 42.100000 | 9.000000 | MNHN --- | OST2 | CHIAA098-15 | |  |
| *P. k. kuhlii* | FR | Haute-Savoie | Gaillard, Allée des Iris 3 | 46.183981 | 6.201323 | MHNG 1958.048 | M1526 | CHIAA013-15 | |  |
| *P. k. kuhlii* | FR | Haute-Savoie | La Roche-sur-Foron | 46.067103 | 6.311864 | MHNG 1981.014 | M1670 | CHIAA025-15 | |  |
| *P. k. kuhlii* | FR | Isère | Isles d'Abeau | 45.616667 | 5.233333 | MNHN --- | 320_123039 | CHIAA090-15 | |  |
| *P. k. kuhlii* | FR | Rhône | Bron | 45.734883 | 4.912076 | MNHN --- | 326_123040 | CHIAA095-15 | |  |
| *P. k. kuhlii* | LI | Cyrenaica | Karkurah | 31.426365 | 20.027860 | NMP 48322 | pb1587 |  | | KM252773.1 |
| *P. k. kuhlii* | LI | Cyrenaica | Tokrah | 32.531763 | 20.572733 | NMP 48326 | pb1591 |  | | KM252774.1 |
| *P. k. kuhlii* | LI | Cyrenaica | Al Abyar | 32.190286 | 20.595832 | NMP 48332 | pb1597 | CHIAA041-15 | |  |
| *P. k. kuhlii* | SW | - | - |  |  | MHNG 1989.023 | M1864 | CHIAA060-15 | |  |
| *P. k. kuhlii* | SW | - | - |  |  | MHNG 1989.024 | M1869 | CHIAA064-15 | |  |
| *P. k. kuhlii* | SW | - | - |  |  | MHNG 1989.025 | M1870 | CHIAA065-15 | |  |
| *P. k. kuhlii* | SW | Bern | Belp | 46.895510 | 7.498312 | NMBE 1058790 |  | CHIAA066-15 | |  |
| *P. k. kuhlii* | SW | Bern | Aarberg | 47.044218 | 7.273278 | NMBE 1063811 |  | CHIAA067-15 | |  |
| *P. k. kuhlii* | SW | Bern | Bern | 46.952137 | 7.444897 | NMBE 1063816 |  | CHIAA068-15 | |  |
| *P. k. kuhlii* | SW | Bern | Bern | 46.952137 | 7.444897 | NMBE 1063819 |  | CHIAA069-15 | |  |
| *P. k. kuhlii* | SW | Genève | Collonge-Bellerive, École de la Californie | 46.240049 | 6.203310 | MHNG 1987.021 | M1802 | CHIAA003-15 | |  |
| *P. k. kuhlii* | SW | Genève | Meyrin, Rue de la Prulay 61 | 46.231187 | 6.075507 | MHNG 1963.076 | M1479 | CHIAA006-15 | |  |
| *P. k. kuhlii* | SW | Genève | Carouge, Rue des Caroubiers 7 | 46.187662 | 6.134435 | MHNG 1969.051 | M1492 | CHIAA007-15 | |  |
| *P. k. kuhlii* | SW | Genève | Vésenaz, Chemin des Rayes 28 | 46.235934 | 6.202837 | MHNG 1969.055 | M1493 | CHIAA008-15 | |  |
| *P. k. kuhlii* | SW | Genève | Malagnou, Parc du Muséum | 46.199001 | 6.158263 | MHNG 1969.057 | M1494 | CHIAA009-15 | |  |
| *P. k. kuhlii* | SW | Genève | Confignon, Chemin de Narly 9 | 46.177777 | 6.090661 |  | M1495 | CHIAA010-15 | |  |
| *P. k. kuhlii* | SW | Genève | Grand-Lancy, Chemin des Champ-Gottreux 13 | 46.174316 | 6.116300 | MHNG 1958.036 | M1524 | CHIAA011-15 | |  |
| *P. k. kuhlii* | SW | Genève | Genève, Rue Calvin 11 | 46.2022 | 6.146932 |  | M1521 | CHIAA012-15 | |  |
| *P. k. kuhlii* | SW | Genève | Genève, Rue de Candolle | 46.198326 | 6.144826 | MHNG 1958.049 | M1527 | CHIAA014-15 | |  |
| *P. k. kuhlii* | SW | Genève | Genève, Place du Bourg-de-Four 1 | 46.200673 | 6.149477 | MHNG 1971.100 | M1577 | CHIAA015-15 | |  |
| *P. k. kuhlii* | SW | Genève | Genève, Hôpital cantonal (H.U.G.) | 46.193372 | 6.149067 | MHNG 1970.100 | M1578 | CHIAA016-15 | |  |
| *P. k. kuhlii* | SW | Genève | Athenaz, Avusy, Route d'Avusy 10A | 46.151818 | 5.994802 | MHNG 1973.057 | M1584 | CHIAA017-15 | |  |
| *P. k. kuhlii* | SW | Genève | Chêne-Bourg, Rue du Gothard 12 | 46.194843 | 6.192002 | MHNG 1959.046 | M1589 | CHIAA018-15 | |  |
| *P. k. kuhlii* | SW | Genève | Chêne-Bourg, Route de Sous-Moulin 16 | 46.192586 | 6.191829 | MHNG 1959.048 | M1590 | CHIAA019-15 | |  |
| *P. k. kuhlii* | SW | Genève | Chêne-Bourg, Avenue du Petit-Senn 41c | 46.192614 | 6.196880 | MHNG 1972.086 | M1586 | CHIAA020-15 | |  |
| *P. k. kuhlii* | SW | Genève | Thônex, Chemin Emile-Bressler 16 | 46.195841 | 6.213381 | MHNG 1981.029 | M1587 | CHIAA021-15 | |  |
| *P. k. kuhlii* | SW | Genève | Chêne-Bourg, Chemin de Floraire 13 | 46.191139 | 6.193380 | MHNG 1959.049 | M1591 | CHIAA022-15 | |  |
| *P. k. kuhlii* | SW | Genève | Arzier, Route des Sendys 1 | 46.460437 | 6.205258 | MHNG 1981.005 | M1667 | CHIAA023-15 | |  |
| *P. k. kuhlii* | SW | Genève | Chêne-Bougeries, Chemin de Grange-Canal 30a | 46.201962 | 6.176786 | MHNG 1981.012 | M1668 | CHIAA024-15 | |  |
| *P. k. kuhlii* | SW | Genève | Genève, Avenue du Bouchet 10 | 46.216332 | 6.120192 | MHNG 1981.006 | M1672 | CHIAA026-15 | |  |
| *P. k. kuhlii* | SW | Genève | Genève, Rue du 31 décembre 22 | 46.203798 | 6.159238 | MHNG 1981.010 | M1677 | CHIAA027-15 | |  |
| *P. k. kuhlii* | SW | Genève | Genève, Rue du Gothard 12 | 46.194852 | 6.191940 | MHNG 1981.008 | M1678 | CHIAA028-15 | |  |
| *P. k. kuhlii* | SW | Genève | Meinier, Chemin du Stade 7b | 46.246656 | 6.231044 | MHNG 1981.009 | M1680 | CHIAA029-15 | |  |
| *P. k. kuhlii* | SW | Genève | Genève, Rue de la Fontaine 9 | 46.201505 | 6.149795 | MHNG 1981.013 | M1681 | CHIAA030-15 | |  |
| *P. k. kuhlii* | SW | Genève | Genève, Rue de la Ferme 14 | 46.188757 | 6.143593 | MHNG 1981.004 | M1684 | CHIAA031-15 | |  |
| *P. k. kuhlii* | SW | Genève | Genève, Rue Rothschild 65 | 46.215941 | 6.146355 | MHNG 1981.007 | M1686 | CHIAA032-15 | |  |
| *P. k. kuhlii* | SW | Genève | Les Acacias, Rue Simon-Durand 9 | 46.190170 | 6.138268 | MHNG 1976.088 | M1690 | CHIAA033-15 | |  |
| *P. k. kuhlii* | SW | Genève | Lancy, Route du Chancy 14 | 46.192478 | 6.121903 | MHNG 1981.055 | M1692 | CHIAA034-15 | |  |
| *P. k. kuhlii* | SW | Genève | Bellevue | 46.252298 | 6.143301 | MHNG 1981.062 | M1703 | CHIAA035-15 | |  |
| *P. k. kuhlii* | SW | Genève | Chêne-Bougeries, Avenue J.-J. Rigaud 3 | 46.203726 | 6.188913 | MHNG 1981.082 | M1707 | CHIAA036-15 | |  |
| *P. k. kuhlii* | SW | Genève | Thônex, Chemin Edouard-Olivet 6 | 46.189567 | 6.202388 | MHNG 1981.084 | M1709 | CHIAA037-15 | |  |
| *P. k. kuhlii* | SW | Genève | Genève, Rue de Bourgogne 16a | 46.210702 | 6.122839 | MHNG 1981.089 | M1714 | CHIAA038-15 | |  |
| *P. k. kuhlii* | SW | Genève | Cointrin, Chemin des Marais 6 | 46.222208 | 6.112276 | MHNG 1981.094 | M1720 | CHIAA046-15 | |  |
| *P. k. kuhlii* | SW | Genève | Genève, Cour des Schtroumpfs | 46.212135 | 6.137594 | MHNG 1981.096 | M1722 | CHIAA047-15 | |  |
| *P. k. kuhlii* | SW | Genève | Genève, Jardin de la Paix, Trembley | 46.219437 | 6.126992 | MHNG 1981.097 | M1723 | CHIAA048-15 | |  |
| *P. k. kuhlii* | SW | Genève | Onex, Collège du Marais, Route de Loëx 22 | 46.184617 | 6.094629 | MHNG 1981.087 | M1712 | CHIAA049-15 | |  |
| *P. k. kuhlii* | SW | Genève | Genève | 46.207602 | 6.113043 | MHNG 1989.026 | M1840 | CHIAA056-15 | |  |
| *P. k. kuhlii* | SW | Genève | Genève, Avenue Calas 16 | 46.189185 | 6.154328 | MHNG 1987.096 | M1849 | CHIAA057-15 | |  |
| *P. k. kuhlii* | SW | Genève | Plan-les-Ouates, Route de Saint-Julien | 46.163961 | 6.106786 | MHNG 1989.027 | M1856 | CHIAA058-15 | |  |
| *P. k. kuhlii* | SW | Genève | Veyrier | 46.166643 | 6.176365 | MHNG 1807.028 | M845 | CHIAA001-15 | |  |
| *P. k. kuhlii* | SW | Genève | Chêne-Bourg | 46.201822 | 6.201525 | MHNG 1828.067 | M916 | ABBWP082-06 | | KM252777.1 |
| *P. k. kuhlii* | SW | Genève | Genève | 46.207602 | 6.113043 | MHNG 1869.032 | M997 | ABBWP093-06 | |  |
| *P. k. kuhlii* | SW | Genève | Immeuble Ernest-Pictet | 46.213365 | 6.124242 | MHNG 1885.089 | M1127 | ABBWP095-06 | |  |
| *P. k. kuhlii* | SW | Genève | Genève, Rue de Lausanne 121 | 46.220824 | 6.149473 | MHNG 1916.061 |  | ABBWP101-06 | | KM252776.1 |
| *P. k. kuhlii* | SW | Genève | Genève | 46.200118 | 6.155982 | MHNG 1940.015 | M1271a | CHIAA004-15 | |  |
| *P. k. kuhlii* | SW | Genève | Thônex | 46.189285 | 6.202794 | MHNG 1685.014 | M1275a | CHIAA005-15 | |  |
| *P. k. kuhlii* | SW | Genève | Genève, Rue du Môle, devant Migros | 46.212201 | 6.149586 | MHNG 1685.018 |  | ABBWP004-06 | |  |
| *P. k. kuhlii* | SW | Genève | Grd-Saconnex, Chemin Bonveur 22a | 46.224044 | 6.113398 | MHNG 1987.093 | M1822 | CHIAA050-15 | |  |
| *P. k. kuhlii* | SW | Genève | Genève, Rue du Grd-Pré 11 | 46.212601 | 6.137369 | MHNG 1987.094 | M1823 | CHIAA051-15 | |  |
| *P. k. kuhlii* | SW | Genève | Genève, Chemin Charles Roluzzi 45 | 46.179930 | 6.147684 | MHNG 1987.095 | M1824 | CHIAA052-15 | |  |
| *P. k. kuhlii* | SW | Ticino | Monte Carasso, Via al Ram 7b | 46.238478 | 9.022595 | MNHL --- | VT_3179 | CHIAA070-15 | |  |
| *P. k. kuhlii* | SW | Ticino | Avegno Gordevio, Gordevio | 46.199994 | 8.749937 | MNHL --- | VT_3239 | CHIAA071-15 | |  |
| *P. k. kuhlii* | SW | Ticino | Caslano, Via Martelli 19 | 45.974447 | 8.882442 | MNHL --- | VT_3240 | CHIAA072-15 | |  |
| *P. k. kuhlii* | SW | Valais | Sion | 46.233300 | 7.350000 | MHNG 1868.075 | M955 | CHIAA002-15 | |  |
| *P. k. kuhlii* | SW | Valais | Châteauneuf | 46.225505 | 7.334329 | MHNG 1868.076 | M959 | ABBWP085-06 | |  |
| *P. k. kuhlii* | SW | Vaud | Nyon | 46.384478 | 6.234813 | MHNG 1989.032 | M1862 | CHIAA059-15 | |  |
| *P. k. kuhlii* | SW | Vaud | Lausanne, Place Chaudron | 46.523608 | 6.625110 | MHNG 1906.049 | M1143 | ABBWP098-06 | | KM252764.1 |
| *P. k. kuhlii* | SW | Vaud | Échallens | 46.640602 | 6.635420 | MHNG 1989.030 | M1830 | CHIAA053-15 | |  |
| *P. k. kuhlii* | SW | Vaud | Chavannes-des-Bois | 46.315147 | 6.133118 | MHNG 1989.031 | M1835 | CHIAA054-15 | |  |
| *P. k. kuhlii* | SW | Vaud | Lausanne | 46.520945 | 6.633732 | MHNG 1989.029 | M1838 | CHIAA055-15 | |  |
| *P. k. kuhlii* | SW | Vaud | Lausanne | 46.520945 | 6.633732 | MHNG 1989.033 | M1865 | CHIAA061-15 | |  |
| *P. k. kuhlii* | SW | Vaud | Eysins | 46.382169 | 6.208119 | MHNG 1989.034 | M1867 | CHIAA062-15 | |  |
| *P. k. kuhlii* | SW | Vaud | Lausanne | 46.520945 | 6.633732 | MHNG 1989.035 | M1868 | CHIAA063-15 | |  |
| *P. k. kuhlii* | SW | Zürich | Watt | 47.443153 | 8.481420 | MHNG 1988.097 | M1983 | CHIAA073-15 | |  |
| *P. k. kuhlii* | SW | Zürich | Winterthur | 47.476898 | 8.781581 | MHNG 1988.098 | M1977 | CHIAA074-15 | |  |
| *P. k. kuhlii* | SW | Zürich | Zürich | 47.373216 | 8.541391 | MHNG 1988.099 | M1979 | CHIAA075-15 | |  |
| *P. k. kuhlii* | SW | Zürich | Zürich | 47.373216 | 8.541391 | MHNG 1988.100 | M1984 | CHIAA076-15 | |  |
| *P. k. kuhlii* | SW | Zürich | Kloten | 47.457112 | 8.581495 | MHNG 1989.096 | M1975 | CHIAA077-15 | |  |
| *P. k. kuhlii* | SW | Zürich | Zürich | 47.411681 | 8.554866 | MHNG 1989.097 | M1980 | CHIAA078-15 | |  |
| *P. k. kuhlii* | SW | Zürich | Hedingen | 47.301119 | 8.450274 | MHNG 1989.098 | M1974 | CHIAA079-15 | |  |
| *P. k. kuhlii* | SW | Zürich | Dübendorf | 47.394108 | 8.617730 | MHNG 1989.099 | M1981 | CHIAA080-15 | |  |
| *P. k. kuhlii* | SW | Zürich | Dinhard | 47.557241 | 8.762956 | MHNG 1989.100 | M1982 | CHIAA081-15 | |  |
| *P. k. kuhlii* | SW | Zürich | Dielsdorf | 47.476086 | 8.454309 | MHNG 1990.070 | M1976 | CHIAA082-15 | |  |
| *P. k. deserti* | LI | Fezzan | Gabrun | 26.803053 | 13.535300 | NMP 48315 | pb1580 | CHIAA039-15 | |  |
| *P. k. deserti* | LI | Fezzan | Germa | 26.529354 | 13.073130 | NMP 48320 | pb1585 | CHIAA040-15 | |  |
| *P. k. deserti* | LI | Fezzan | Al Fjayj | 26.542120 | 13.310900 | NMP 48321 | pb1586 |  | | KM252758.1 |
| *P. k. deserti* | LI | Fezzan | Gabrun | 26.803053 | 13.535295 | NMP 48304 | pb1569 | CHIAA042-15 | |  |
| *P. k. deserti* | LI | Fezzan | Gabrun | 26.803053 | 13.535295 | NMP 48305 | pb1570 | CHIAA043-15 | |  |
| *P. k. deserti* | LI | Fezzan | Gabrun | 26.803053 | 13.535295 | NMP 48313 | pb1578 | CHIAA044-15 | |  |
| *P. k. deserti* | LI | Fezzan | Gabrun | 26.803053 | 13.535295 | NMP 48314 | pb1579 | CHIAA045-15 | |  |
| *P. k. deserti* | MO | - | Oued Drâa | 29.595401 | -8.305178 | NMP 90058 | pb2671 |  | | KM252759.1 |
| *P. k. deserti* | MO | - | Oued Drâa | 29.595401 | -8.305178 | NMP 90059 | pb2672 |  | | KM252760.1 |
| *P. k. deserti* | MO | Meknès-Tafilalet | Tamtattouchte | 31.676558 | -5.539602 | NMP 90071 | pb2684 | CHIAA091-15 | |  |
