# Supplementary material for: Two Mitochondrial Barcodes for one Biological Species: The Case of European Kuhl's Pipistrelles (Chiroptera)
Source: PLoS One. 2015 Aug 4;10(8):e0134881. doi: 10.1371/journal.pone.0134881 (PMC4524706; doi:10.1371/journal.pone.0134881)
Supplement: S2 File — (DOCX) [file pone.0134881.s002.docx]

S2 File. GenBank accession numbers for the cyt-*b* sequences used in the Fig. 3.

Hapl. 1: EU360658.; Hapl. 2: HQ687494.1, KM252777.1, KM252776.1, JX566947.1, JX566941.1, JX566935.1, JX566934.1, JX566932.1, JX566930.1, JX566927.1, EU360657.1, EU360656.1, EU360655.1, DQ120848.1, DQ120845.1, DQ120844.1, DQ120843.1, DQ120842.1, DQ120841.1, AJ426619.1; Hapl. 3: EU360654.1; Hapl. 4: KC520772.1; Hapl. 5: KC520774.1; Hapl. 6: KC520773.1; Hapl. 7: KC146390.1; Hapl. 8: KC146389.1, KC146388.1, KM252775.1, KM252772.1, KM252771.1, KM252770.1, KM252769.1, KM252768.1, KM252767.1, KM252766.1, KF498639.1, KC684548.1, KC684547.1, KC684546.1, KF218394.1, KF218393.1, KF218391.1, KF218390.1, AJ504445.1; Hapl. 9: KF218392.1; Hapl. 10: KM252773.1; Hapl. 11: KM252774.1; Hapl. 12: KF218395.1; Hapl. 13: HQ687496.1; Hapl. 14: HQ687493.1; Hapl. 15: KM252758.1, KM252757.1, KM252756.1; Hapl. 16: HQ687497.1, HQ687495.1, KM252765.1, KM252764.1, KM252763.1, KM252762.1, EU360660.1, EU360659.1, EU360653.1, EU360650.1, DQ120847.1, AJ504444.1, KM252760.1; Hapl. 17: DQ120846.1; Hapl. 18: EU360651.1; Hapl. 19: EU360652.1; Hapl. 20: KM252761.1, AJ426609.1, AJ426608.1, AJ426607.1, KM252759.1; *P. pygmaeus*: JX566938.1, JX566937.1; *P. pipistrellus*: KF874521.1
