# Supplementary material for: Two Mitochondrial Barcodes for one Biological Species: The Case of European Kuhl's Pipistrelles (Chiroptera)
Source: PLoS One. 2015 Aug 4;10(8):e0134881. doi: 10.1371/journal.pone.0134881 (PMC4524706; doi:10.1371/journal.pone.0134881)
Supplement: S3 File — (DOCX) [file pone.0134881.s003.docx]

S3 File. Microsatellite primer informations and multiplexes. All amplifications were performed with the following PCR cycling: initial denaturation step for 15 min at 95 °C, followed by 32 cycles with a denaturation for 40 s at 94 °C, annealing for 90 s (temperature indicated in the table below) and extension for 60 s at 72 °C, and a final extension for 30 min at 60 °C.

| Locus name | Approximate fragment length | Sequence forward | Sequence reverse | Annealing temperature | Multiplex number | Dye | Original publication | Alleles number  Europe/North Africa (Both) |
| --- | --- | --- | --- | --- | --- | --- | --- | --- |
| EF6 | 160-190 bp | ATCACATTTTTGAAGCAT | ATCTGTTTTTCTCTCCTTAT | 55-50 °C | 1 | DY751 (black) | [[39](#_ENREF_39)] | 12/8 (12) |
| L45 | 90-120 bp | GGAATGCAGCAATGTTAGGT | AAGGGCAGTGACAGGAAAGC | 65-60 °C | 3 | DY751 (black) | [[42](#_ENREF_42)] | 13/8 (13) |
| Paur05 | 220-250 bp | GGACAGTATGCCATGTTATGCTG | GCACTTTCACAAACCTAGATGG | 65-60 °C | 2 | DY751 (black) | [[40](#_ENREF_40)] | 9/3 (11) |
| Ppip05 | 160-200 bp | GTTGACTCTGTTCGGGAAAGGAG | TGGGACTAAGACACAAGCCTGG | 65-60 °C | 3 | BMN6 (green) | [[41](#_ENREF_41)] | 18/1 (19) |
| Ppip06 | 110-150 bp | TGCCCAACCAAGTGAGCTACAG | GCTCCAGTGTTGACTTTCCTCTCTC | 56 °C | 4 | BMN6 (green) | [[41](#_ENREF_41)] | 15/9 (24) |
