# Supplementary material for: Two Mitochondrial Barcodes for one Biological Species: The Case of European Kuhl's Pipistrelles (Chiroptera)
Source: PLoS One. 2015 Aug 4;10(8):e0134881. doi: 10.1371/journal.pone.0134881 (PMC4524706; doi:10.1371/journal.pone.0134881)
Supplement: S4 File — (DOCX) [file pone.0134881.s004.docx]

S4 File. Multilocus genotypes of all specimens. List of the 111 vouchered samples of Kuhl’s Pipistrelles with their voucher number (if any), and field number, as in SI 1. The next columns represent the two alleles genotyped for the five microsatellite loci.

| Scientific number | Field number | EF6 | | Paur05 | | L45 | | Ppip05 | | Ppip06 | |
| --- | --- | --- | --- | --- | --- | --- | --- | --- | --- | --- | --- |
| MNHN --- | 321_122386 | 162 | 162 | 233 | 242 | 106 | 114 | 185 | 187 | 128 | 146 |
| MNHN --- | 323_122384 | 176 | 178 | 237 | 241 | 100 | 110 | 181 | 182 | 132 | 138 |
| MNHN --- | 324_122385 | 174 | 178 | 231 | 233 | 106 | 112 | 184 | 187 | 140 | 140 |
| MNHN --- | 322_124776 | 162 | 180 | 231 | 231 | 110 | 112 | 186 | 186 | 136 | 150 |
| MNHN --- | 306_STF20 | 162 | 172 | 237 | 239 | 112 | 116 | 184 | 186 | 128 | 130 |
| MNHN --- | 307_STF21 | 162 | 176 | 239 | 239 | 96 | 108 | 184 | 184 | 136 | 140 |
| MNHN --- | 314_STF24 | 162 | 176 | 239 | 239 | 96 | 96 | 184 | 184 | 132 | 136 |
| MNHN --- | 313_STF25 | 162 | 178 | 239 | 239 | 96 | 110 | 190 | 190 | 136 | 142 |
| MNHN --- | STF26 | 162 | 162 | 233 | 239 | 112 | 118 | 184 | 184 | 138 | 138 |
| MNHN --- | STF27 | 162 | 180 | 239 | 239 | 96 | 106 | 177 | 190 | 134 | 142 |
| MNHN --- | STF28 | 162 | 180 | 239 | 239 | 96 | 102 | 184 | 184 | 128 | 138 |
| MNHN --- | 346_PAI1 | 162 | 162 | 239 | 239 | 106 | 110 | 190 | 190 | 140 | 140 |
| MNHN --- | FLO1 | 178 | 182 | 237 | 239 | 96 | 110 | 185 | 188 | 130 | 140 |
| MNHN --- | OST2 | 162 | 162 | 233 | 233 | 96 | 96 | 185 | 186 | 136 | 136 |
| MHNG 1958.048 | M1526 | 162 | 176 | 231 | 235 | 96 | 98 | 182 | 188 | 142 | 146 |
| MHNG 1981.014 | M1670 | 174 | 178 | 231 | 231 | 96 | 108 | 177 | 177 | 134 | 142 |
| MNHN --- | 320_123039 | 174 | 174 | 231 | 231 | 100 | 110 | 188 | 188 | 130 | 138 |
| MNHN --- | 326_123040 | 174 | 186 | 233 | 239 | 110 | 112 | 184 | 185 | 130 | 140 |
| NMP 48322 | pb1587 | 178 | 178 | 227 | 227 | 108 | 108 | 165 | 165 | 139 | 143 |
| NMP 48326 | pb1591 | 180 | 180 | 227 | 227 | 100 | 108 | 165 | 165 | 139 | 139 |
| NMP 48332 | pb1597 | 168 | 176 | 227 | 229 | 96 | 112 | 165 | 165 | 137 | 143 |
| MHNG 1989.023 | M1864 | 178 | 180 | 231 | 239 | 110 | 110 | 184 | 184 | 140 | 144 |
| MHNG 1989.024 | M1869 | 162 | 180 | 239 | 239 | 100 | 102 | 183 | 183 | 134 | 144 |
| MHNG 1989.025 | M1870 | 178 | 180 | 231 | 235 | 102 | 102 | 180 | 191 | 134 | 154 |
| NMBE 1058790 |  | 162 | 184 | 237 | 237 | 102 | 102 | 184 | 186 | 132 | 142 |
| NMBE 1063811 |  | 162 | 178 | 237 | 239 | 96 | 102 | 188 | 188 | 136 | 146 |
| NMBE 1063816 |  | 162 | 162 | 237 | 239 | 104 | 106 | 176 | 177 | 140 | 142 |
| NMBE 1063819 |  | 162 | 176 | 239 | 239 | 102 | 104 | 182 | 184 | 142 | 142 |
| MHNG 1987.021 | M1802 | 162 | 168 | 237 | 239 | 106 | 112 | 182 | 185 | 144 | 144 |
| MHNG 1963.076 | M1479 | 162 | 162 | 237 | 239 | 100 | 106 | 182 | 190 | 134 | 138 |
| MHNG 1969.051 | M1492 | 162 | 162 | 237 | 245 | 106 | 108 | 187 | 188 | 142 | 144 |
| MHNG 1969.055 | M1493 | 178 | 180 | 231 | 233 | 104 | 104 | 186 | 187 | 134 | 136 |
| MHNG 1969.057 | M1494 | 162 | 176 | 233 | 239 | 102 | 108 | 180 | 186 | 144 | 150 |
|  | M1495 | 162 | 176 | 239 | 239 | 106 | 110 | 187 | 189 | 144 | 144 |
| MHNG 1958.036 | M1524 | 162 | 178 | 231 | 237 | 106 | 110 | 187 | 189 | 136 | 136 |
|  | M1521 | 162 | 178 | 235 | 239 | 100 | 110 | 182 | 186 | 142 | 144 |
| MHNG 1958.049 | M1527 | 162 | 174 | 231 | 231 | 100 | 100 | 177 | 186 | 134 | 152 |
| MHNG 1971.100 | M1577 | 176 | 180 | 235 | 237 | 100 | 112 | 173 | 188 | 134 | 142 |
| MHNG 1970.100 | M1578 | 162 | 178 | 235 | 239 | 110 | 110 | 186 | 188 | 136 | 136 |
| MHNG 1973.057 | M1584 | 162 | 178 | 231 | 237 | 102 | 110 | 182 | 184 | 138 | 144 |
| MHNG 1959.046 | M1589 | 176 | 180 | 237 | 239 | 104 | 106 | 185 | 188 | 144 | 150 |
| MHNG 1959.048 | M1590 | 162 | 180 | 231 | 237 | 102 | 106 | 182 | 187 | 136 | 142 |
| MHNG 1972.086 | M1586 | 162 | 170 | 239 | 239 | 100 | 112 | 182 | 182 | 136 | 142 |
| MHNG 1981.029 | M1587 | 176 | 176 | 235 | 237 | 100 | 106 | 177 | 188 | 134 | 142 |
| MHNG 1959.049 | M1591 | 162 | 186 | 233 | 237 | 96 | 110 | 184 | 189 | 130 | 146 |
| MHNG 1981.005 | M1667 | 162 | 178 | 235 | 237 | 88 | 110 | 186 | 187 | 132 | 136 |
| MHNG 1981.012 | M1668 | 180 | 180 | 231 | 241 | 96 | 108 | 182 | 186 | 136 | 140 |
| MHNG 1981.006 | M1672 | 162 | 178 | 229 | 231 | 102 | 104 | 186 | 187 | 130 | 144 |
| MHNG 1981.010 | M1677 | 162 | 172 | 239 | 241 | 110 | 112 | 173 | 186 | 136 | 138 |
| MHNG 1981.008 | M1678 | 176 | 176 | 233 | 235 | 102 | 108 | 177 | 187 | 136 | 148 |
| MHNG 1981.009 | M1680 | 180 | 180 | 231 | 235 | 100 | 110 | 181 | 187 | 136 | 142 |
| MHNG 1981.013 | M1681 | 176 | 186 | 233 | 237 | 106 | 110 | 180 | 187 | 134 | 146 |
| MHNG 1981.004 | M1684 | 178 | 180 | 231 | 235 | 110 | 110 | 183 | 184 | 112 | 144 |
| MHNG 1981.007 | M1686 | 178 | 186 | 231 | 239 | 100 | 112 | 187 | 188 | 138 | 140 |
| MHNG 1976.088 | M1690 | 162 | 178 | 233 | 241 | 100 | 108 | 175 | 189 | 130 | 140 |
| MHNG 1981.055 | M1692 | 162 | 178 | 235 | 239 | 106 | 110 | 186 | 190 | 134 | 138 |
| MHNG 1981.062 | M1703 | 178 | 178 | 235 | 239 | 100 | 100 | 173 | 188 | 138 | 152 |
| MHNG 1981.082 | M1707 | 178 | 182 | 231 | 231 | 96 | 110 | 177 | 188 | 136 | 138 |
| MHNG 1981.084 | M1709 | 162 | 174 | 231 | 233 | 96 | 106 | 184 | 189 | 138 | 142 |
| MHNG 1981.089 | M1714 | 178 | 182 | 233 | 237 | 106 | 110 | 180 | 189 | 136 | 140 |
| MHNG 1981.094 | M1720 | 174 | 178 | 233 | 237 | 100 | 108 | 186 | 187 | 138 | 152 |
| MHNG 1981.096 | M1722 | 162 | 180 | 233 | 235 | 108 | 108 | 180 | 188 | 136 | 146 |
| MHNG 1981.097 | M1723 | 162 | 174 | 233 | 237 | 96 | 108 | 187 | 188 | 140 | 144 |
| MHNG 1981.087 | M1712 | 162 | 164 | 237 | 237 | 112 | 112 | 182 | 183 | 136 | 148 |
| MHNG 1989.026 | M1840 | 174 | 186 | 235 | 239 | 108 | 108 | 184 | 190 | 140 | 144 |
| MHNG 1987.096 | M1849 | 162 | 174 | 235 | 239 | 104 | 104 | 178 | 183 | 142 | 152 |
| MHNG 1989.027 | M1856 | 186 | 186 | 239 | 239 | 110 | 112 | 183 | 187 | 138 | 144 |
| MHNG 1807.028 | M845 | 174 | 178 | 231 | 237 | 106 | 112 | 184 | 187 | 142 | 144 |
| MHNG 1828.067 | M916 | 162 | 182 | 233 | 235 | 100 | 100 | 186 | 189 | 134 | 146 |
| MHNG 1869.032 | M997 | 180 | 182 | 231 | 239 | 100 | 108 | 177 | 184 | 136 | 142 |
| MHNG 1885.089 | M1127 | 162 | 176 | 231 | 239 | 112 | 112 | 182 | 182 | 140 | 146 |
| MHNG 1916.061 |  | 174 | 178 | 237 | 239 | 100 | 104 | 177 | 177 | 130 | 136 |
| MHNG 1940.015 | M1271a | 176 | 182 | 229 | 237 | 100 | 110 | 177 | 186 | 142 | 150 |
| MHNG 1685.014 | M1275a | 180 | 180 | 237 | 239 | 110 | 112 | 186 | 190 | 138 | 138 |
| MHNG 1685.018 |  | 162 | 162 | 237 | 239 | 106 | 108 | 182 | 182 | 142 | 150 |
| MHNG 1987.093 | M1822 | 178 | 178 | 237 | 237 | 96 | 102 | 180 | 190 | 136 | 146 |
| MHNG 1987.094 | M1823 | 174 | 178 | 237 | 239 | 96 | 108 | 193 | 193 | 138 | 140 |
| MHNG 1987.095 | M1824 | 162 | 182 | 237 | 239 | 110 | 110 | 177 | 186 | 136 | 146 |
| MNHL --- | VT_3179 | 162 | 182 | 237 | 237 | 102 | 102 | 182 | 184 | 144 | 148 |
| MNHL --- | VT_3239 | 162 | 162 | 237 | 239 | 104 | 106 | 180 | 180 | 136 | 136 |
| MNHL --- | VT_3240 | 162 | 174 | 237 | 239 | 106 | 108 | 175 | 175 | 130 | 146 |
| MHNG 1868.075 | M955 | 162 | 174 | 237 | 239 | 96 | 108 | 175 | 184 | 136 | 138 |
| MHNG 1868.076 | M959 | 162 | 178 | 231 | 239 | 102 | 106 | 184 | 184 | 136 | 138 |
| MHNG 1989.032 | M1862 | 162 | 162 | 233 | 239 | 100 | 100 | 183 | 186 | 138 | 152 |
| MHNG 1906.049 | M1143 | 162 | 180 | 237 | 237 | 102 | 110 | 187 | 187 | 138 | 144 |
| MHNG 1989.030 | M1830 | 162 | 182 | 239 | 239 | 96 | 110 | 175 | 182 | 134 | 150 |
| MHNG 1989.031 | M1835 | 174 | 178 | 239 | 239 | 88 | 88 | 177 | 180 | 140 | 144 |
| MHNG 1989.029 | M1838 | 176 | 178 | 239 | 239 | 106 | 110 | 180 | 184 | 130 | 138 |
| MHNG 1989.033 | M1865 | 162 | 164 | 231 | 235 | 96 | 112 | 175 | 187 | 130 | 146 |
| MHNG 1989.034 | M1867 | 172 | 180 | 231 | 241 | 88 | 102 | 180 | 188 | 132 | 144 |
| MHNG 1989.035 | M1868 | 178 | 180 | 235 | 241 | 96 | 108 | 173 | 182 | 136 | 142 |
| MHNG 1988.097 | M1983 | 162 | 162 | 235 | 239 | 96 | 102 | 175 | 186 | 136 | 140 |
| MHNG 1988.098 | M1977 | 162 | 174 | 231 | 237 | 96 | 112 | 186 | 187 | 128 | 138 |
| MHNG 1988.099 | M1979 | 162 | 180 | 233 | 239 | 104 | 104 | 175 | 186 | 134 | 140 |
| MHNG 1988.100 | M1984 | 162 | 186 | 237 | 237 | 104 | 104 | 181 | 182 | 140 | 140 |
| MHNG 1989.096 | M1975 | 162 | 178 | 237 | 239 | 102 | 102 | 186 | 186 | 140 | 140 |
| MHNG 1989.097 | M1980 | 162 | 162 | 231 | 237 | 96 | 106 | 180 | 184 | 138 | 140 |
| MHNG 1989.098 | M1974 | 162 | 180 | 237 | 239 | 102 | 112 | 175 | 184 | 142 | 142 |
| MHNG 1989.099 | M1981 | 162 | 162 | 237 | 239 | 102 | 106 | 185 | 185 | 138 | 140 |
| MHNG 1989.100 | M1982 | 162 | 174 | 233 | 237 | 102 | 106 | 184 | 184 | 142 | 148 |
| MHNG 1990.070 | M1976 | 180 | 180 | 233 | 241 | 106 | 112 | 180 | 186 | 136 | 136 |
| NMP 48315 | pb1580 | 174 | 176 | 223 | 227 | 96 | 100 | 165 | 165 | 137 | 137 |
| NMP 48320 | pb1585 | 174 | 180 | 227 | 227 | 96 | 104 | 165 | 165 | 123 | 143 |
| NMP 48321 | pb1586 | 180 | 180 | 227 | 227 | 100 | 100 | 165 | 165 | 125 | 139 |
| NMP 48304 | pb1569 | 176 | 180 | 227 | 227 | 100 | 104 | 165 | 165 | 135 | 135 |
| NMP 48305 | pb1570 | 180 | 184 | 227 | 227 | 102 | 102 | 165 | 165 | 137 | 139 |
| NMP 48313 | pb1578 | 180 | 180 | 227 | 227 | 100 | 102 | 165 | 165 | 129 | 137 |
| NMP 48314 | pb1579 | 178 | 180 | 227 | 227 | 96 | 102 | 165 | 165 | 133 | 137 |
| NMP 90058 | pb2671 | 172 | 184 | 227 | 227 | 98 | 112 | 165 | 165 | 139 | 139 |
| NMP 90059 | pb2672 | 176 | 176 | 227 | 227 | 96 | 106 | 165 | 165 | 135 | 141 |
| NMP 90071 | pb2684 | 176 | 182 | 227 | 227 | 96 | 98 | 165 | 165 | 137 | 139 |
