# Supplementary material for: Two Mitochondrial Barcodes for one Biological Species: The Case of European Kuhl's Pipistrelles (Chiroptera)
Source: PLoS One. 2015 Aug 4;10(8):e0134881. doi: 10.1371/journal.pone.0134881 (PMC4524706; doi:10.1371/journal.pone.0134881)

S5 File. Population structure inferred for K=2 to 8 by the Bayesian clustering analysis. This analysis is based on 5 nuclear loci and 111 individuals genotyped. Individuals carrying the Western mitochondrial lineage are placed to the left of the median bar, while those carrying the Eastern mitochondrial lineage are displayed on the right. The 13 North African Kuhl’s pipistrelles appear in the rightmost part of the graph. See Fig. 5 for further details about the Structure analysis parameters.


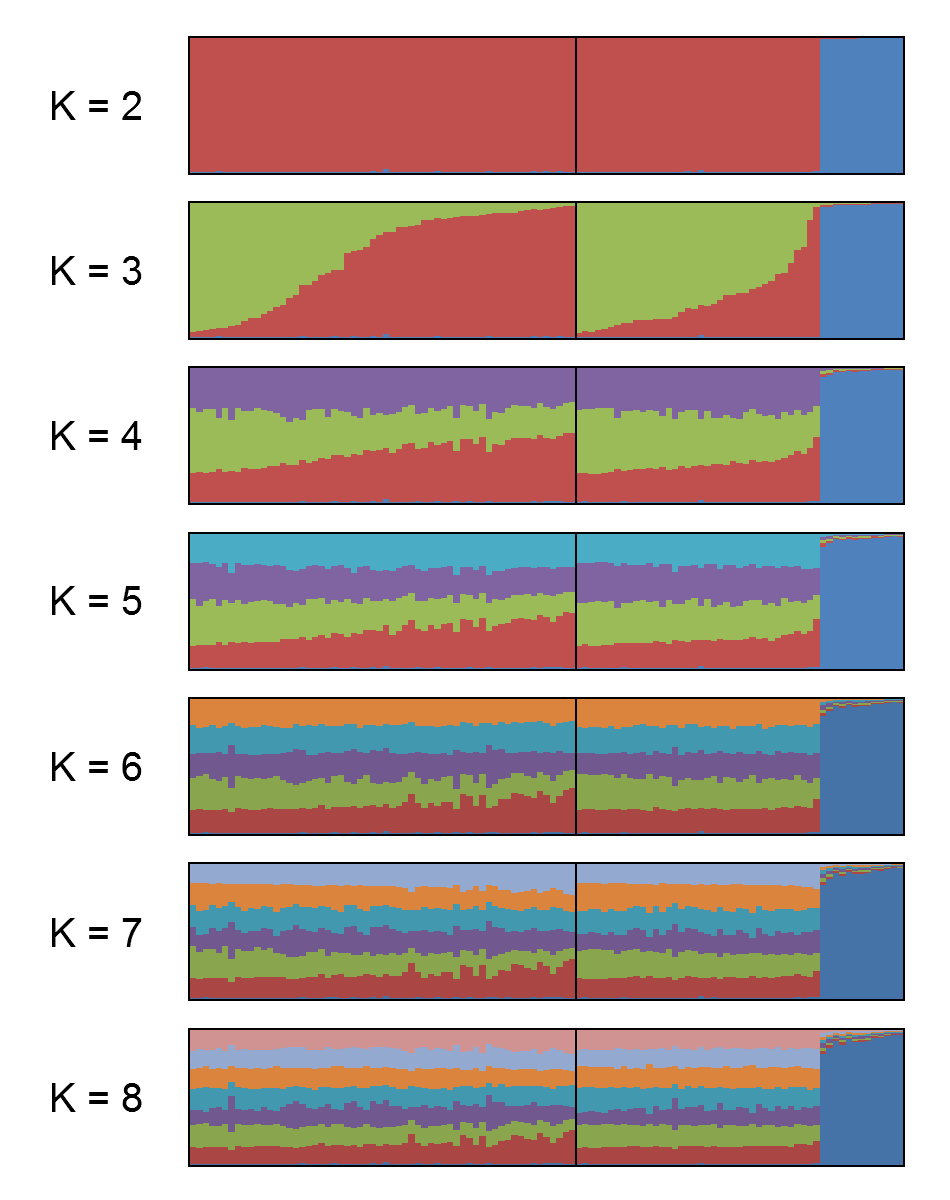

Supplement: S5 File — (DOCX) [file pone.0134881.s005.docx]
